# Artificial Intelligence for Radiation Oncology Applications Using Public Datasets

**DOI:** 10.1016/j.semradonc.2022.06.009

**Published:** 2022-10

**Authors:** Kareem A. Wahid, Enrico Glerean, Jaakko Sahlsten, Joel Jaskari, Kimmo Kaski, Mohamed A. Naser, Renjie He, Abdallah S.R. Mohamed, Clifton D. Fuller

**Affiliations:** * Department of Radiation Oncology, The University of Texas MD Anderson Cancer Center, Houston, Texas, USA; † Department of Neuroscience and Biomedical Engineering, Aalto University School of Science, Espoo, Finland; ‡ Department of Computer Science, Aalto University School of Science, Espoo, Finland

## Abstract

Artificial intelligence (AI) has exceptional potential to positively impact the field of radiation oncology. However, large curated datasets - often involving imaging data and corresponding annotations - are required to develop radiation oncology AI models. Importantly, the recent establishment of Findable, Accessible, Interoperable, Reusable (FAIR) principles for scientific data management have enabled an increasing number of radiation oncology related datasets to be disseminated through data repositories, thereby acting as a rich source of data for AI model building. This manuscript reviews the current and future state of radiation oncology data dissemination, with a particular emphasis on published imaging datasets, AI data challenges, and associated infrastructure. Moreover, we provide historical context of FAIR data dissemination protocols, difficulties in the current distribution of radiation oncology data, and recommendations regarding data dissemination for eventual utilization in AI models. Through FAIR principles and standardized approaches to data dissemination, radiation oncology AI research has nothing to lose and everything to gain.

## Introduction

The rise of artificial intelligence (AI) and machine learning in recent years has seen an explosion in technical approaches to statistical modeling, driven mostly by innovation on large datasets in the computational science community. These groundbreaking efforts have benefitted immensely from the availability of large-scale highly annotated datasets, such as the ImageNet visual recognition task dataset,^1^ or the Modified National Institute of Standards and Technology (MNIST) handwriting dataset.^2^ These datasets have allowed researchers to train and benchmark AI algorithms using transparent and comparable methods through a known corpus with defined properties at large-scale, thereby substantially accelerating AI growth. In fact, some have purported that the modern AI age can be traced to the convolutional neural network AlexNet winning the ImageNet challenge in 2012;^3^ clearly, this task could not have been accomplished without the careful construction and curation of the ImageNet platform several years earlier.^4^ During the same interval, the “Big Cancer Data” era was arguably initiated by the availability of large-scale public cancer databases. Specifically, The Cancer Genome Atlas (TCGA) has had a transformative effect on oncologic research.^5^ Crucial for the oncologic imaging community, and consequently the radiation oncology community, a subsidiary effort, The Cancer Imaging Archive (TCIA),^6^ was originally developed as a repository for matched image data from TCGA cases; however, TCIA has become a leader in its own right for distributing reliable and high-quality datasets independent of TCGA. Subsequently, radiation oncology has now entered a new era for AI applications due to an increasing deluge of data afforded by resources such as TCIA. In the following sections, we review and discuss the role of data sharing in the radiation oncology ecosystem, important regulatory and ethical considerations, and contemporary databases available for public investigation.

## FAIR Data Publication as a Normative Component of Modern Scientific Dissemination and Knowledge Generation as a Coherent Process

**Figure 1:**
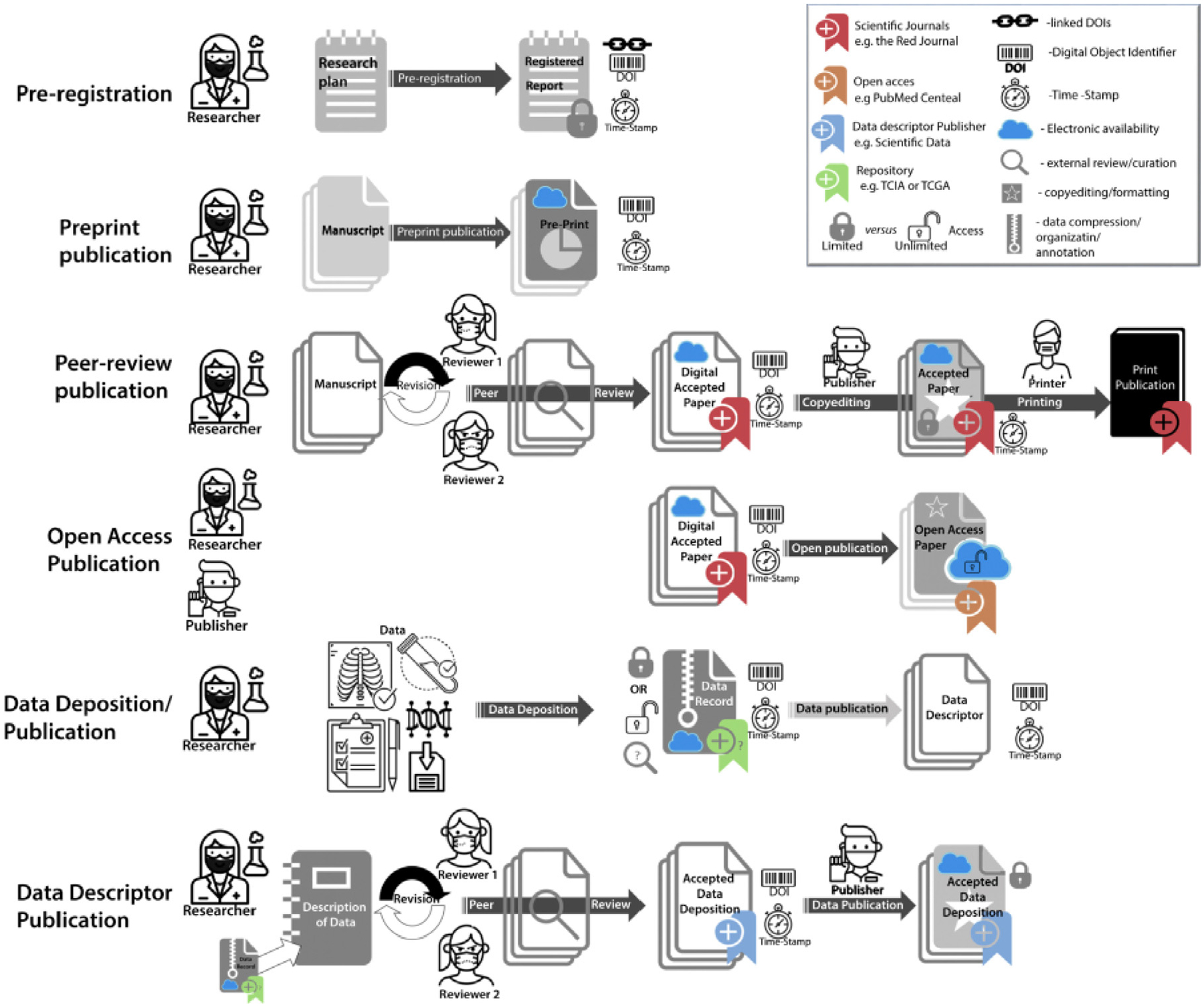
Graphical representation of current independent steps in scientific dissemination process. Reprinted from Fuller et al.^9^

Importantly, the availability of big data on its own does not ensure the development of robust, reproducible, and clinically impactful datasets. This is particularly true in medical domains such as radiation oncology, where data can be scarce, heterogeneous, and often non-standardized.^7^ To ensure the usefulness of large datasets and foster data re-usability, it is fundamental to follow the Findable, Accessible, Interoperable, Reusable (FAIR) principles for scientific data management.^8^ These principles emphasize machine-actionability, that is, the capacity of computational systems to find, access, interoperate, and reuse data with minimal human intervention. To foster the widespread implementation of FAIR scientific principles in day-to-day scientific workflows, several steps can and should be taken by practitioners of open-science practices in the radiation oncology community. These practices should include the utilization of study preregistration, manuscript preregistration, open-access journals, and code/data sharing (Fig. 1). We briefly review these components here; an in-depth discussion on these concepts can be found in greater detail in Fuller et al.^9^

Scientific research is often plagued by widespread biases, for example, “p-hacking” (repeat measurements leading to significant findings),^10^ among others.^11^ Study preregistration serves as a tool to potentially reduce these pitfalls.^12^ The concept of preregistration involves specifying details of a research plan on a registry, such as through the Open Science Framework,^13^ before performing the research study. Notably, the practice of preregistration has been shown to increase analytical rigor and increase the publication of null findings.^14^

After study completion, the utilization of preprints, a manuscript that precedes formal peer review, allows for rapid dissemination of study findings to the general public.^15^ Drawing inspiration from the well-known physics preprint server arXiv, in 2019 a clinical medicine preprint repository, medRxiv, was established.^16^ Since its inception, medRxiv has quickly become a leading figure in medically related preprint dissemination.^17^ The benefits of publishing preprints for radiation oncology are numerous, chief among which include near-immediate distribution of scientific findings, ease of use, circumvention of journal politics and dominant narratives, and complete access by the general public. Naturally, the use of preprints raises the potential for faulty analysis. However, these concerns seem minor compared to the relative benefit of rapid dissemination of information which could then be verified through indicators of internal validity and wider scientific community evaluation. Closely related to concepts regarding preprints is the use of open-access options for scientific manuscripts. By allowing wider distribution of scientific findings, open-access options may lead to a greater return on investment for scientific innovation.^18^ As of 2008, the National Institutes of Health (NIH) has mandated publicly funded research be made available through PubMed Central.^19^ Moreover, as of 2021, the Plan S initiative will require that all EU-funded efforts be published through open access journals or platforms.^20^ These geopolitical pushes highlight the importance of open access models for manuscript dissemination and will likely be a core staple of future radiation oncology research.

Finally, data and code accessibility, which are particularly germane to AI-driven analyses in radiation oncology, form a more recently established central tenant for disseminating scientific information. An in-depth discussion of data repositories is described in other sections of this review (see “NCI Policy, Vision, and Supported Repositories” and “TCIA as a Model for Effective FAIR Data in Oncology”). Moreover, the need for structured data and corresponding annotations has prompted the rise of journals dedicated to publishing data and/or corresponding records explaining dataset contents, termed “data descriptors”.^21^ Examples of journals publishing data descriptors include Medical Physics and Nature Scientific Data.^22^ Finally, code repositories, such as GitHub, have allowed for the rapid and dynamic development of code related to scientific studies in the medical domain, and fostered community engagement for future developments.^23^

**Figure 2:**
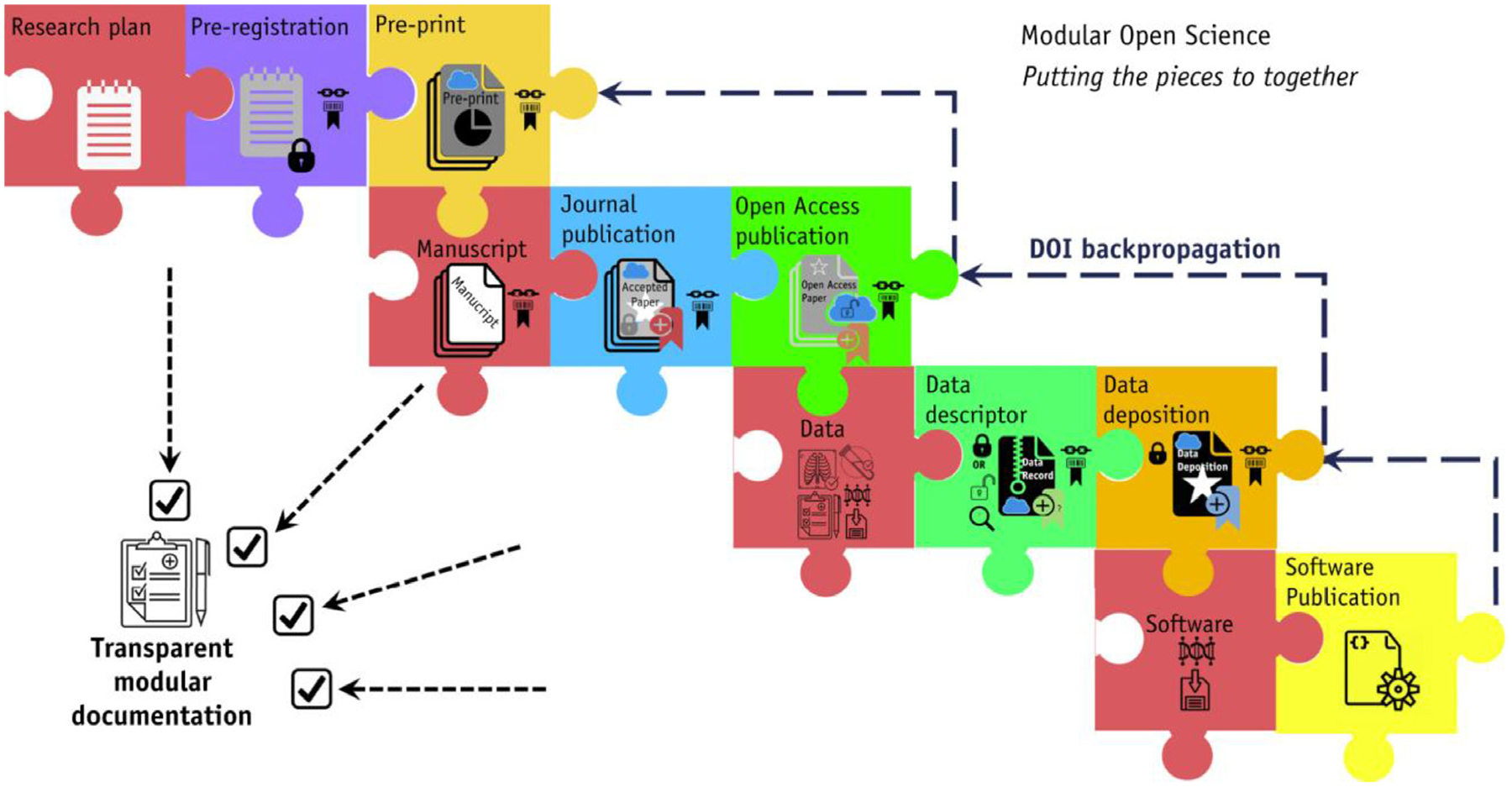
Proposed transparent modular scientific dissemination process, using metadata or digital object identifier (DOI) to link individual processes. Reprinted from Fuller et al.^9^

While the current components of scientific dissemination remain relatively independent, in the future, one could envision a modular framework where automated processes link these components in an integrated fashion (Fig. 2). Corresponding checklists ensuring proper completion of steps could allow for the routine integration of these components in scientific workflows. Through these processes, the quality of scientific dissemination would increase, which could have great potential for advances in radiation oncology research.

## Annotation/Ontology Considerations for Public Datasets

Broadly, there are two main types of data relevant to AI model development using public datasets in radiation oncology - image data formats and image annotation formats. The majority of hospital picture archiving and communication system infrastructure stores medical images in Digital Imaging and Communications in Medicine (DICOM) format, which is the recognized international standard for image data storage.^24^ For ease of distribution, DICOM data is often transformed into Neuroimaging Informatics Technology Initiative format, which is increasingly seen as a standard for reproducible imaging research.^25^ While image data is often standardized and distributed relatively easily due to established nomenclature and customs, unfortunately, image annotations are not often stored in a single common format.^26^ For example, image annotations can be stored in picture archiving and communication system and other systems as DICOM presentation state objects which may vary from vendor to vendor. The recently developed DICOM structured reporting (DICOM-SR) standard has allowed to somewhat combat this lack of standardization in annotation but there still remains problems with intersite variability of annotation templates.^27^ For non-graphic annotations, the annotation and image markup format was recently developed and incorporated into DICOM-SR.^28^ Within radiation oncology, the DICOM radiotherapy (DICOM-RT) standard has allowed for a systematic characterization of radiotherapy-related data.^29^ However, DICOM-RT was not developed with AI/machine-learning applications in mind, thus annotations derived from these files can at times be challenging to incorporate directly into models; community-driven undertakings have developed some solutions to these problems.^30,31^ Thus, ongoing efforts will be needed to reach a consensus on the best practices to standardize image annotation data for radiation oncology applications.

Outside of file objects and corresponding metadata standardization, there are additional considerations for radiation oncology annotation data that are crucial for public distribution. Specifically, nomenclature conventions can often vary across institutions. For example, nomenclature for target/non-target structures is often highly variable (Table 1). Subsequently, standardized nomenclatures applied to targets, normal tissue structures, and treatment planning concepts and metrics would allow for the more facile integration of radiation oncology data for standardized distribution and use in AI models. Therefore, from 2015–2018, the American Association of Physicists in Medicine (AAPM) collated a group of experts in imaging, radiation oncology, and machine learning (Task Group 263), to provide radiation oncology nomenclature guidelines for use in clinical trials, data-pooling initiatives, population-based studies, and routine clinical care.^32,33^ Among the many accomplishments of AAPM Task Group 263, the group was able to standardize: (1) structure names across image processing and treatment planning system platforms, (2) nomenclature for dosimetric data, (3) templates for clinical trial groups, and (4) formalisms for nomenclature schema which could accommodate the addition of other structures defined in the future. Through these guidelines, annotation processes for radiation oncology datasets and corresponding public data deposition should be more easily standardized for use in reproducible and robust research applications.

## International Differences in Privacy Guidelines

In research integrity and ethics, it is accepted across public, health, and research institutions around the world that the privacy of the study participants must always be respected and never compromised to avoid concrete risks such as identity theft, blackmailing, or any other possible adverse consequences that a subject might face if their identity is disclosed with the data. Institutional review boards and ethical committees ensure that scientific research follows the highest standards to protect the safety of human subjects. However, ethics is not law, and while the ethical review process is comparable across countries, the legal principles and interpretation of data protection vary considerably and even deter FAIR data reuse. Currently in the USA, the Health Insurance Portability and Accountability Act (HIPAA) advises the removal of 18 pieces of protected health information (PHI) when sharing de-identified data^34^ (Table 2). By removing this PHI, human subject data in the USA can be considered “anonymous” or at least “anonymous enough” so that their data is considered to not fall under HIPAA and can then be easily reused for scientific research. However, concerns have been raised regarding the potential for intricate biomedical data that could be used for re-identification purposes, for example, brain fingerprinting^35^ and sophisticated algorithmic methods for re-identifying supposedly anonymous individuals. With these concerns in mind, the European Commission has worked on the General Data Protection Regulation (GDPR) which was unveiled in May 2018. GDPR’s main goal is to protect data subjects by ensuring that organizations respect and protect the personal data associated with an individual. While GDPR’s main goal is aligned with research ethics to enable full data protection of the data subject, its strict guidelines have however been perceived at times by the scientific community as an impediment to scientific progress.^36,37^ The current conundrum of having FAIR data that should also adhere to GDPR is potentially solvable with improved techniques for de-identification as well as other approaches like differential privacy and federated analysis.^38^

## Considerations for Anonymization, De-identification and Privacy-Enhancement (e.g., “De-facing”) for Public Datasets

Chief among concerns for utilizing patient-derived medical data in public datasets is proper anonymization and de-identification of PHI. The terms de-identification and anonymization, when applied to medical data can encompass a wide array of definitions, and at times can be vague, inconsistent, or even contradictory.^39^ Often, data are said to be anonymized when PHI has been completely removed such that the data can no longer be associated with an individual in any manner, while de-identification refers to the general removal of PHI.^40^ More technical definitions include de-identification referring to rule-based techniques to remove PHI, with anonymization referring to statistical/probabilistic techniques to remove PHI.^39^ For the purposes of this review, we will refer to the terms de-identification and anonymization interchangeably.

For obvious pieces of medical information, such as patient name, medical record number, date of birth, and other demographics, the anonymization process is often straightforward in removing this data or meta-data from associated files. DICOM formatted files typically contain metadata that links images to information regarding patient demographics and image acquisition parameters, among other information.^41^ In cases where meta-data is available in DICOM or similar files, these data can be stripped using commonly available tools, such as the Radiological Society of North America Clinical Trial Processor,^42^ though care should still be taken to ensure all data has been properly modified. Moreover, PHI may be “burned” into existing images, such as when considering radiographs directly scanned into electronic medical records. These embedded pieces of information can often be removed using optical character recognition techniques or similar methods; however, they can provide additional challenges in ensuring the full removal of text-based PHI.^43^

**Figure 3:**
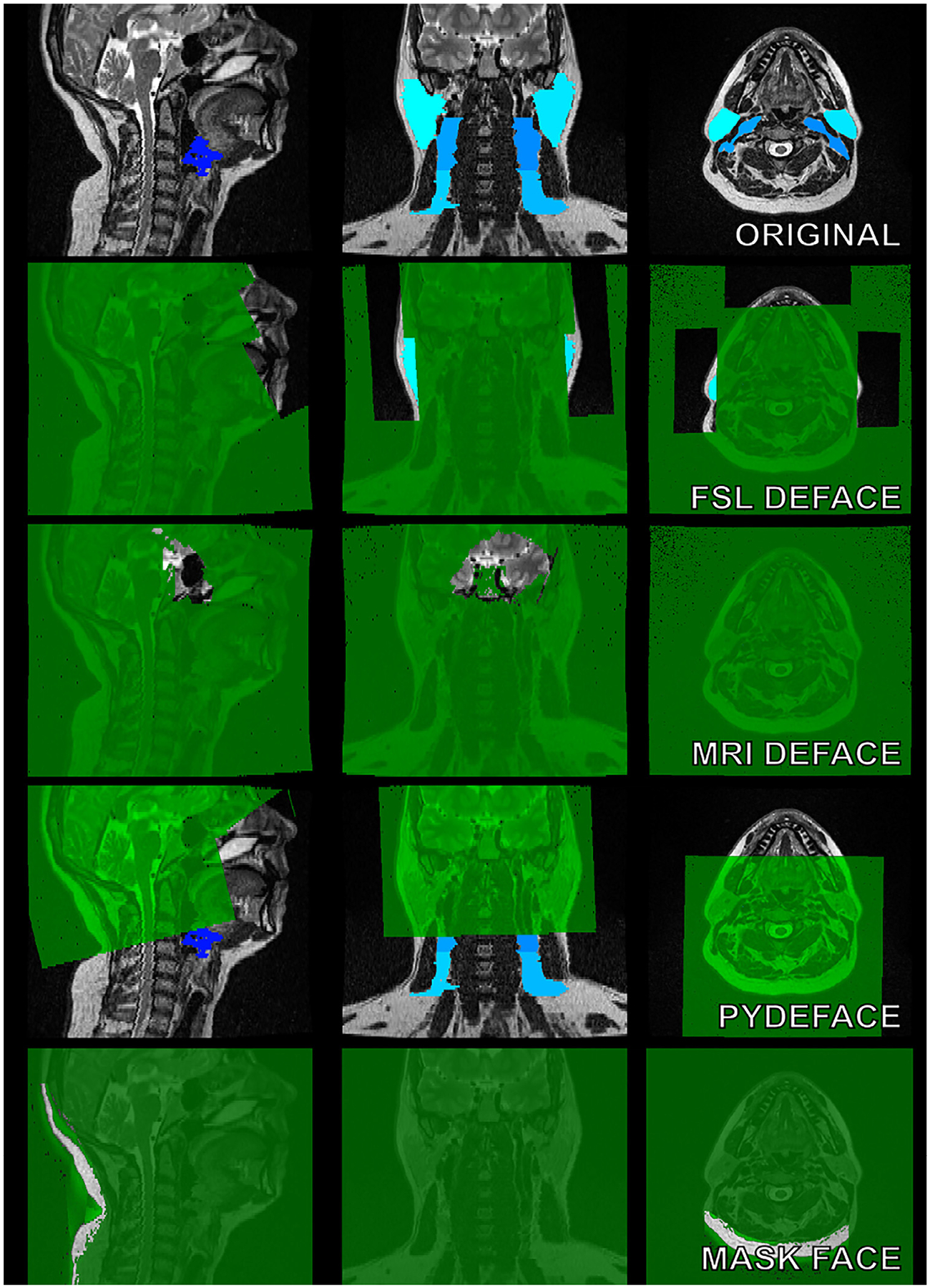
Comparison of MRI de-facing tools. We tested the performance of 4 state-of-the-art tools for face de-identification. On the top row, the original T2-weighted MRI with tissue annotations (lymph node levels, glands) are shown in blue. Masks that each tool automatically creates to remove facial structures from the image volume are shown in green. These tools are popular within the neuroimaging community and were designed for defacing MRIs while preserving brain structures. In many cases, tissues of interest for radiation oncology applications are obscured or removed through the use of these tools.

Perhaps the subject of most controversy within the domain of publicly releasing patient medical image data concerns imaging data that contains readily identifiable facial features. HIPAA references “full-face photographs and any comparable images” as a part of PHI. This raises specific concerns for high-resolution images where the intricacies of facial features can be reconstructed (i.e., CT scans, MRI, etc.) to generate similar “comparable” visualizations of a patient’s face with relative ease. Several studies have shown the potential danger in releasing unaltered medical images containing facial features as they can often be easily recognized by humans or machines.^44–46^ For example, using facial recognition software paired to MRI-derived facial reconstructions, 83% of research participants were able to be identified from their MRI scan.^46^ Within the realm of radiation oncology, perhaps the avenue where the greatest amount of concern for facial identification is in the generation of public medical imaging datasets of head and neck cancer. While brain images are often processed such that obvious facial features are removed (i.e., skull stripping), these crude pre-processing techniques will likely remove important information for building predictive models with head and neck cancer imaging data, prohibiting their use in data resharing strategies. “De-facing” tools, where voxels that correspond to the areas of the patient’s facial features are either removed or altered are one solution. However, they encounter the problem of information loss in important areas needed for predictive modeling or treatment planning. While several studies have investigated the effects of de-facing for applications in neuroimaging,^47–50^ to our knowledge only one study has investigated the effects of de-facing tools for radiation oncology applications in head and neck cancer.^51^ As an example use case, we demonstrate organs at risk are obscured with 4 state-of-the-art de-facing tools^49,52–54^ (Fig. 3), which would have obvious downstream consequences for analysis. Importantly, Sahlsten et al. highlight that the specific selection of de-facing methods has a significant impact on the radiotherapy organs at risk for AI algorithmic development, in some cases rendering structures completely unusable.^51^ While existing tools are seemingly unsatisfactory, future de-facing approaches based on deep learning may be a promising solution.^55^

## NCI Policy, Vision, and Supported Repositories

Broadly, the sharing of biomedical data generated through research studies allows the scientific community to expedite the translation of findings into knowledge, products, and procedures to improve health.^56^ As of 2003, the NIH has implemented a Data Sharing Policy which encourages data to “be made as widely and freely available as possible while safeguarding the privacy of participants, and protecting con-fidential and proprietary data.” Specifically, investigators were required to specify data sharing protocols within NIH grant applications. These protocols for data sharing could be accomplished through the use of data archives, data enclaves, or under the auspices of the principal investigator. Congruent with the increasing ability to generate, store, share, and combine data, in 2015 the NIH initiated a more comprehensive data sharing policy in tandem with efforts to modernize data sharing infrastructure in its Plan for Increasing Access to Scientific Publications and Digital Scientific Data from NIH Funded Scientific Research.^57^ Effective in 2023, the NIH has issued a new Final NIH Policy for Data Management and Sharing which will require NIH funded researchers to prospectively submit a plan detailing how scientific data and metadata will be managed and shared taking into account potential restrictions or limitations^58^; this plan will replace the 2003 NIH Data Sharing Policy. These policies underscore the importance of data stewardship and management for nationally-funded research, and have influenced data sharing practices to funders of radiation oncology research, particularly the National Cancer Institute (NCI).

Within the Final NIH Policy for Data Management and Sharing, detailed documentation has been provided to help researchers select specific repositories for data deposition (“Selecting a Repository for Data Resulting from NIH-Supported Research”). Ideally, where applicable, data should be deposited in discipline or data-type specific repositories. The NIH has provided a list of approved specific repositories at https://www.nlm.nih.gov/NIHbmic/domain_specific_repositories.html; a subset of radiation oncology related repositories is shown in Table 3. For an in-depth discussion on one specific NIH-approved data repository particularly salient for radiation oncology applications, we refer the reader to the section of our review titled “TCIA as a Model for Effective FAIR Data in Oncology”. When no appropriate disciplinary or data-type specific repositories are available, the NIH recommends the use of generalist or institutional repositories. For example, Figshare is an appropriate and well-established generalist repository that can permanently store datasets and assigns DOIs to all published research items.^59^

**Figure 4:**
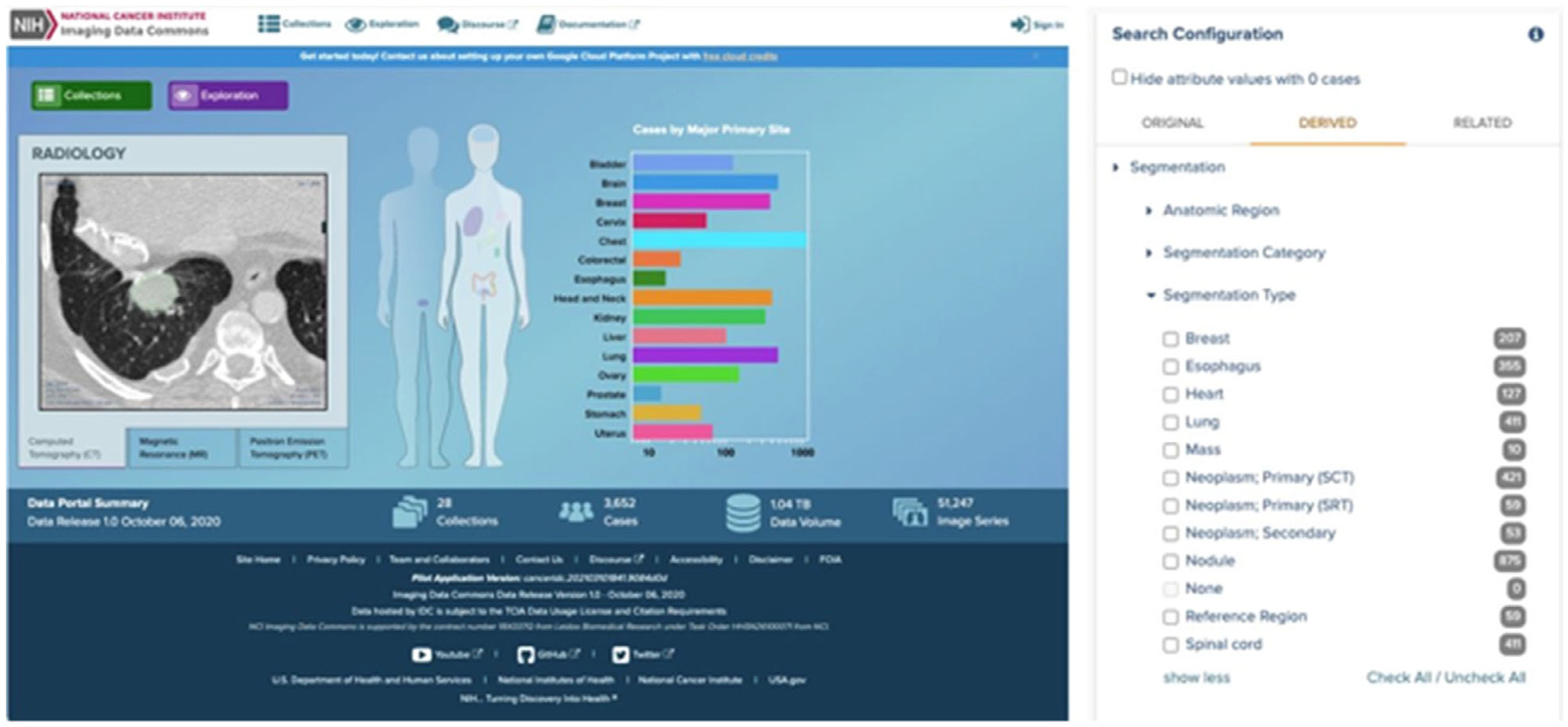
Example of imaging data commons (IDC) portal user interface. Reprinted from Fedorov et al.^62^

Driven by improvements and innovations in cloud-computing paradigms for use in big data research, the NCI has created the Cancer Research Data Commons (CRDC) as a component of a national cancer data ecosystem.^60^ The NCI CRDC includes cloud-based domain-specific data repositories and analysis-focused cloud resources to facilitate collaborative and standardized research practices using diverse data types. Within the CRDC, several data repositories have been established, such as the Genomic Data Commons^61^ and the Imaging Data Commons.^62^ As opposed to previously described data repositories, the cloud-based infrastructure of the CRDC allows researchers to utilize data in real-time without the need for local downloading of data files. Moreover, through these data repositories, disparate data sources (imaging, genomic, proteomic, clinical trial, etc.) can be combined and investigated with compute resources provided by cloud environments, thereby providing researchers with the ability to perform robust harmonized analysis. An example of the IDC user portal interface is shown in Figure 4. The integrated analysis capabilities provided by infrastructure such as CRDC will likely have a significant impact on radiation oncology research in coming years.

## TCIA as a Model for Effective FAIR Data in Oncology

**Figure 5:**
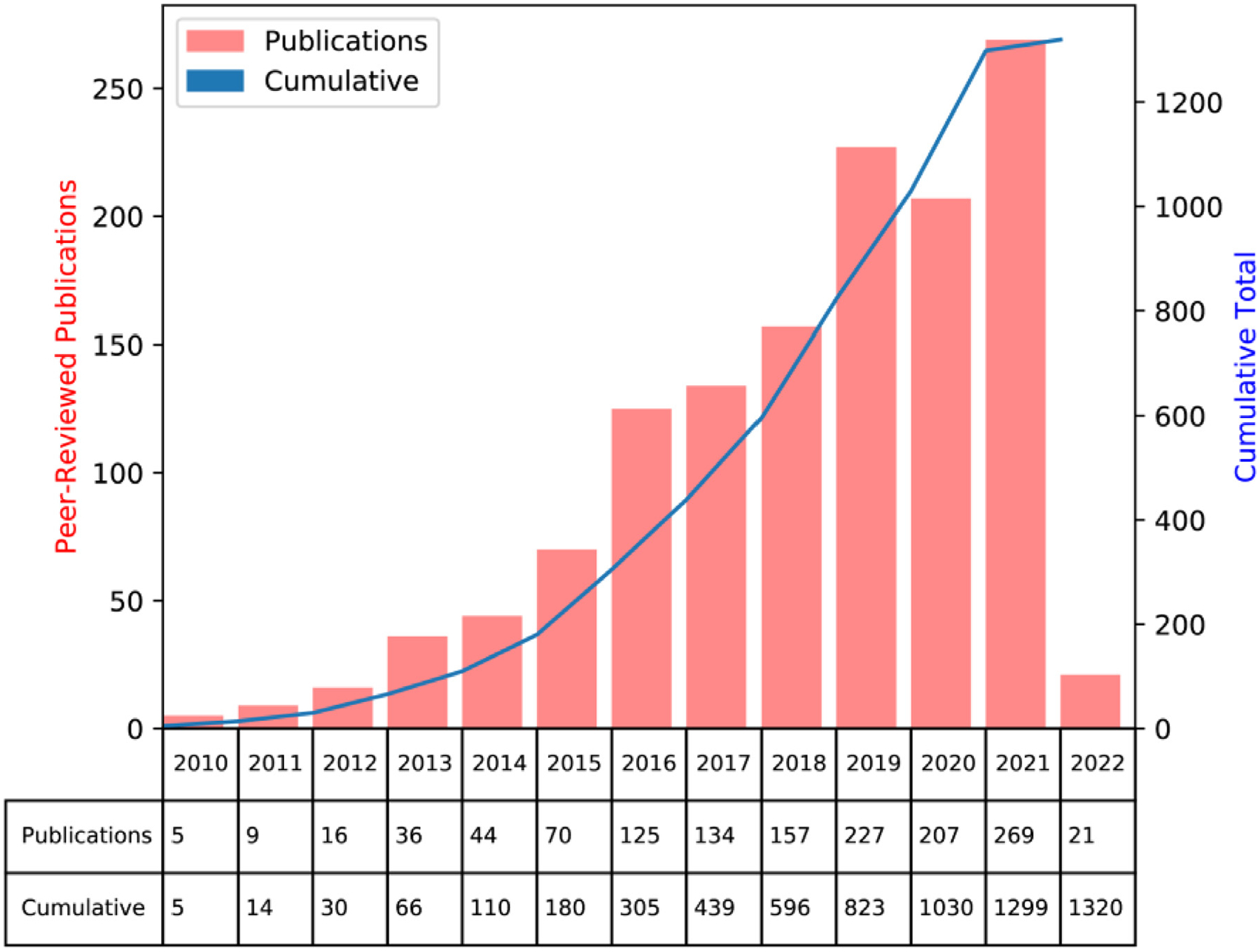
Publications over time related to the cancer imaging archive (TCIA) databases. Graph generated from TCIA website (https://www.cancerimagingarchive.net/publications/) on February 18, 2022.

TCIA is a recently developed service that de-identifies and hosts various medical imaging datasets and supporting data for public distribution. Launched in 2011 with funding from the NCI,^6^ this integrated database has offered the imaging community large volumes of curated data for exploratory image analysis, computational model development, and model validation.^63^ Provided human data originates from various sources, ranging from small-scale calibration studies to large-scale clinical trials. These imaging data have been crucial to developing contemporary medical AI models and catapulted TCIA as a de-facto leader of medical image data dissemination (Fig. 5). It stands to reason that TCIA will continue to provide much-needed high-quality datasets for clinical decision support tool development in the coming years, particularly for radiation oncology.

The overarching structure of the TCIA is stratified into individual collections defined by a common disease (e.g., brain cancer, head-neck cancer, lung cancer), imaging modality (e.g., MRI, CT, PET, histopathology), and/or research focus. Collections are assigned persistent digital object identifiers (DOIs), thereby allowing researchers to reference and acquire datasets.^64^ DOIs contain “Primary Data”, that is, radiological or pathological images, which can be coupled to supporting data (demographics, clinical outcomes, annotations, genomic information, etc.). Before publication, datasets are rigorously curated to ensure acceptable image quality and data integrity. Currently, TCIA utilizes the Posda open-source framework^65^ to aid in the curation process and remove any identifying information in metadata. Collection contents are described through “wiki pages”, which also list relevant publications and instructions for data use. Increasingly, focused “data descriptors,” in-depth manuscripts detailing individual datasets, such as those published through Nature Scientific Data,^22^ are also generated for TCIA collections to engender greater transparency in data generation, collection protocols, and intended use-cases. For end-users, TCIA provides web interfaces and software (National Biomedical Imaging Archive Data Retriever) to easily retrieve and catalog collections on local computing infrastructure.

TCIA data is most often available through standardized imaging formats such as DICOM.^24^ Importantly, TCIA has formed a corpus for not only raw imaging data, but also corresponding supporting data, such as region of interest segmentations through DICOM radiotherapy structure set (RTSTRUCT) files and clinical outcome data. Therefore, TCIA houses a rich stream of information for supervised machine learning segmentation and classification models. For radiation oncology applications, DICOM radiotherapy plan (RTPLAN) and DICOM radiotherapy dose (RTDOSE) are often also included in collections which can be used for model development germane to radiation therapy planning. A list of currently available TCIA collections that include RTPLAN and/or RTDOSE data is shown in Table 4. Currently, most collections with corresponding radiotherapy planning data correspond to head and neck cancer. Importantly, this subset of data comes with additional important considerations for re-use (discussed more in the “Considerations for Anonymization, De-identification and Privacy-Enhancement (e.g., “De-facing”) for Public Datasets” section). As TCIA continues to engender straightforward integration of community contributions, the number of collections that include radiotherapy-related data is expected to continue to rapidly increase over time.

## Examples of Published “Challenges” Using Public Datasets

Data “challenges,” that is, competitions where datasets are publicly provided to interested participants to solve a specific problem, have been a staple in developing modern-day cutting-edge AI algorithms. For example, the ImageNet Large Scale Visual Recognition Challenge, a competition where participants are tasked with classifying photographic images of common objects, was the impetus for the rise of deep learning approaches for computer vision applications.^66^ A similar trend has emerged in the medical domain, particularly for medical imaging, where anonymized data is provided to challenge participants to solve important healthcare problems.^67,68^ The field of radiation oncology, which is heavily centered on image-based workflows, is no exception to this increasing trend, with several radiotherapy-related data challenges emerging in recent years. Here, we summarize a few key data challenges that have been particularly impactful for radiation oncology applications.

**Figure 6:**
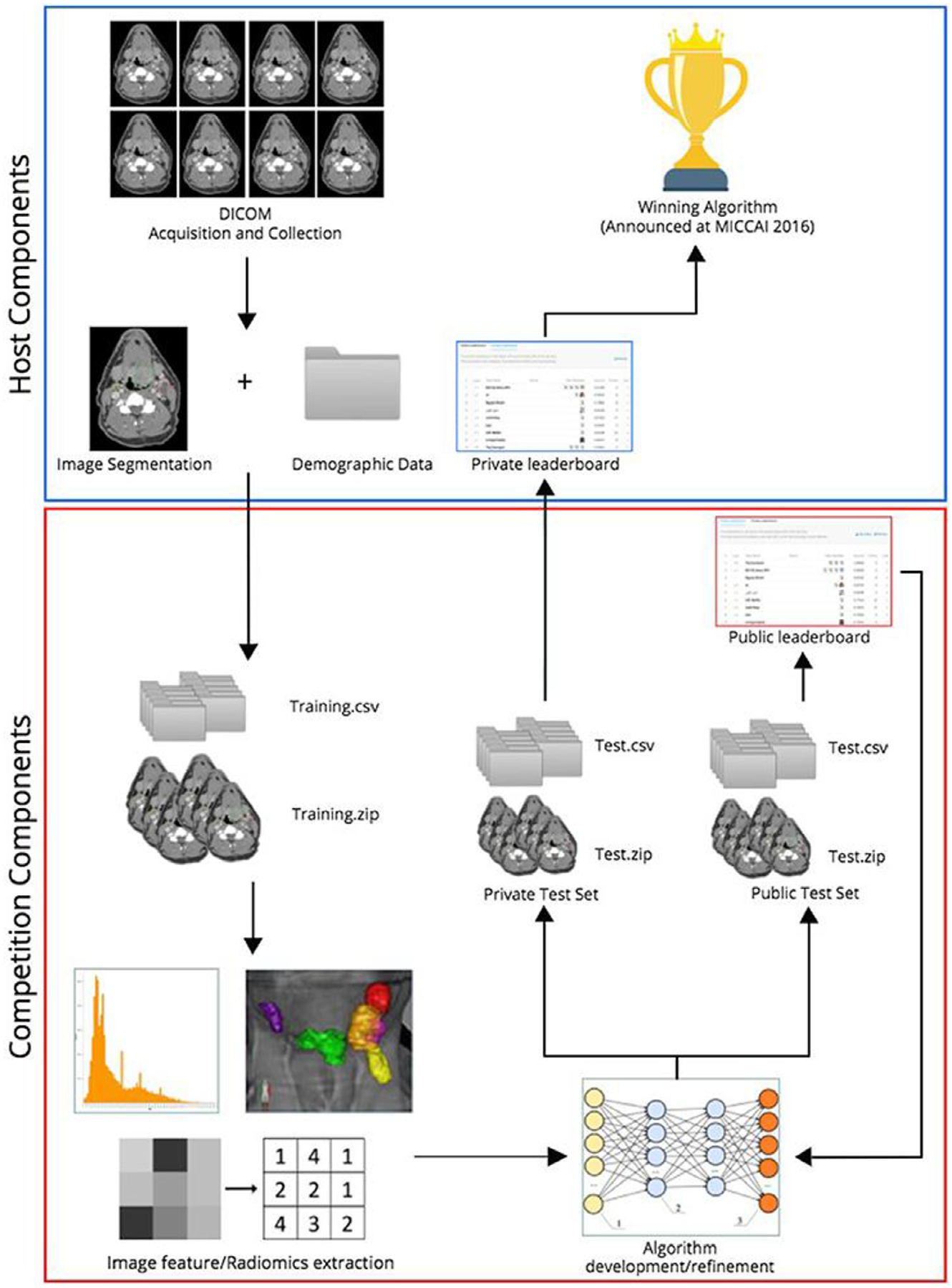
Overview of 2016 oropharynx cancer (OPC) radiomics challenge. Reprinted from Elhalawani et al.^70^

In 2016, inspired by the up-and-coming trend of radiomics, i.e., the use of quantitative features derived from medical imaging,^69^ the University of Texas MD Anderson Cancer Center and the Medical Image Computing and Computer Assisted Intervention Society organized two public radiomics challenges in the head and neck radiation oncology domain.^70^ Through the Kaggle InClass commercial educationally-oriented platform,^71^ the organizers tasked participants to develop predictive models to: (1) classify patients based on human papillomavirus (HPV) status, and (2) predict local tumor recurrence status. A large number of contrast-enhanced CT images of oropharyngeal cancer patients and corresponding clinical data^72^ were provided to participants to build models to solve the 2 tasks through evaluation of independent test data (Fig. 6). The majority of participants used pre-defined radiomic features extracted from images in combination with machine learning models to solve the 2 challenges. Many participants also utilized the provided clinical data in constructing their models. Interestingly, the winner of the HPV classification challenge only utilized radiomic features, while the winner of the recurrence prediction challenge only utilized clinical features. While the challenge has not been renewed for additional iterations, these important results highlighted the difficulty of integrating imaging and clinical data not only for designing data challenges in radiation oncology but also for eventual downstream model implementation.

**Figure 7:**
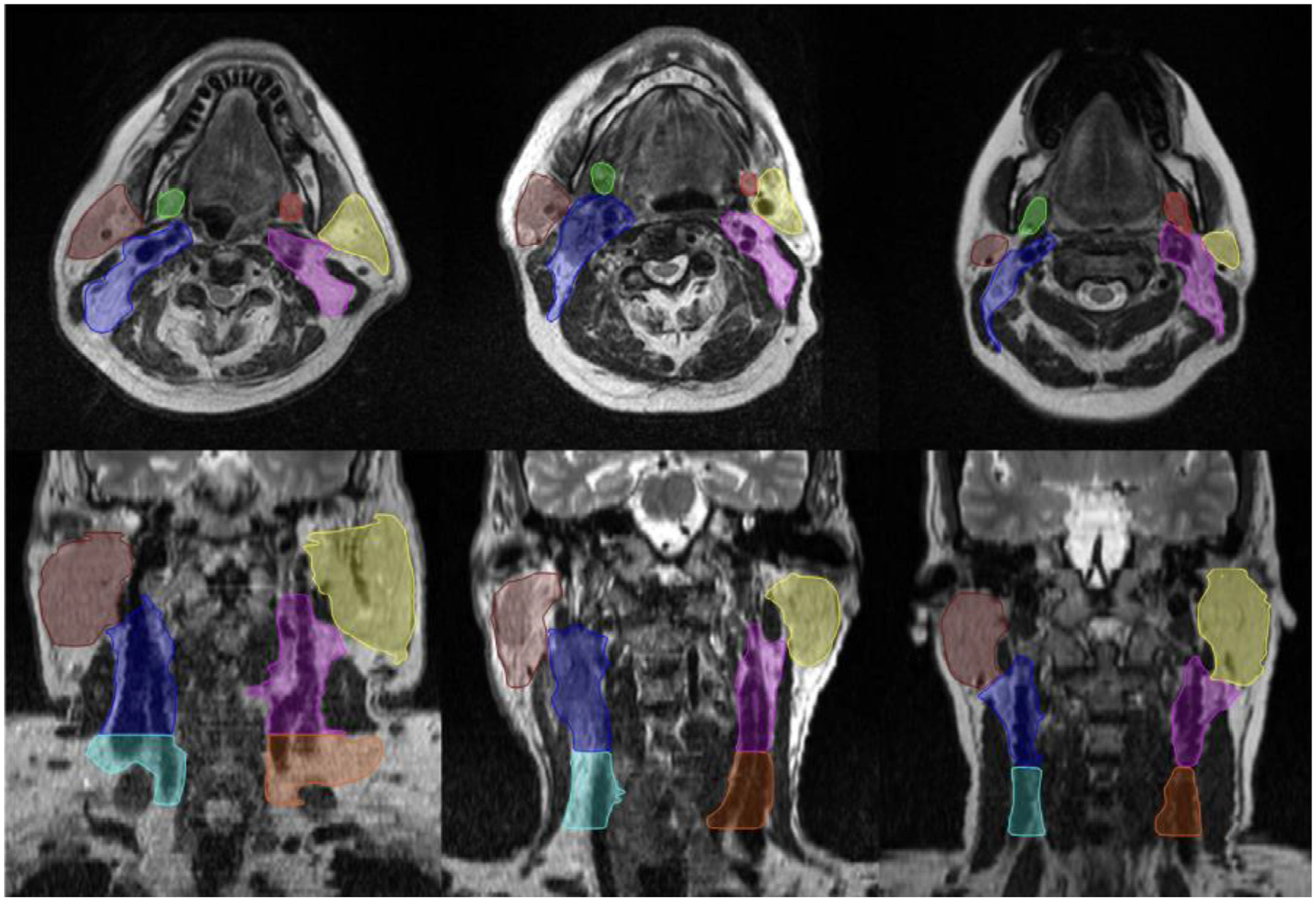
Contoured structure [left submandibular gland (red), right submandibular gland (green), left parotid gland (yellow), right parotid gland (brown), left lymph node level II (blue), right lymph node level II (pink), left lymph node level III (orange), right lymph node level III (light-blue)] for 2019 “RT-MAC” challenge. Reprinted from Cardenas et al.^73^

In 2019, the AAPM hosted a 2-part competition, titled the “RT-MAC” challenge, for auto-segmentation of radiotherapy-related structures using MRI scans.^73^ Participants were tasked with developing algorithms to segment parotid glands, submandibular glands, and various lymph node levels (Fig. 7). The challenge used a relatively limited number of training cases (*n* = 35), but participants were still able to generate segmentation results of reasonable quality on the independent pre-AAPM challenge test set (*n* = 10) and online challenge test set (*n* = 10). These datasets have become publicly available in their entirety through TCIA,^73^ allowing for the community to continue improving upon methods for radiotherapy planning segmentation. Moreover, given the rapidly increasing interest in MRI-guided radiotherapy,^74^ it is foreseeable that analogous datasets could be released in the near future to aid in adaptive radiotherapy auto-segmentation applications.

**Figure 8.**
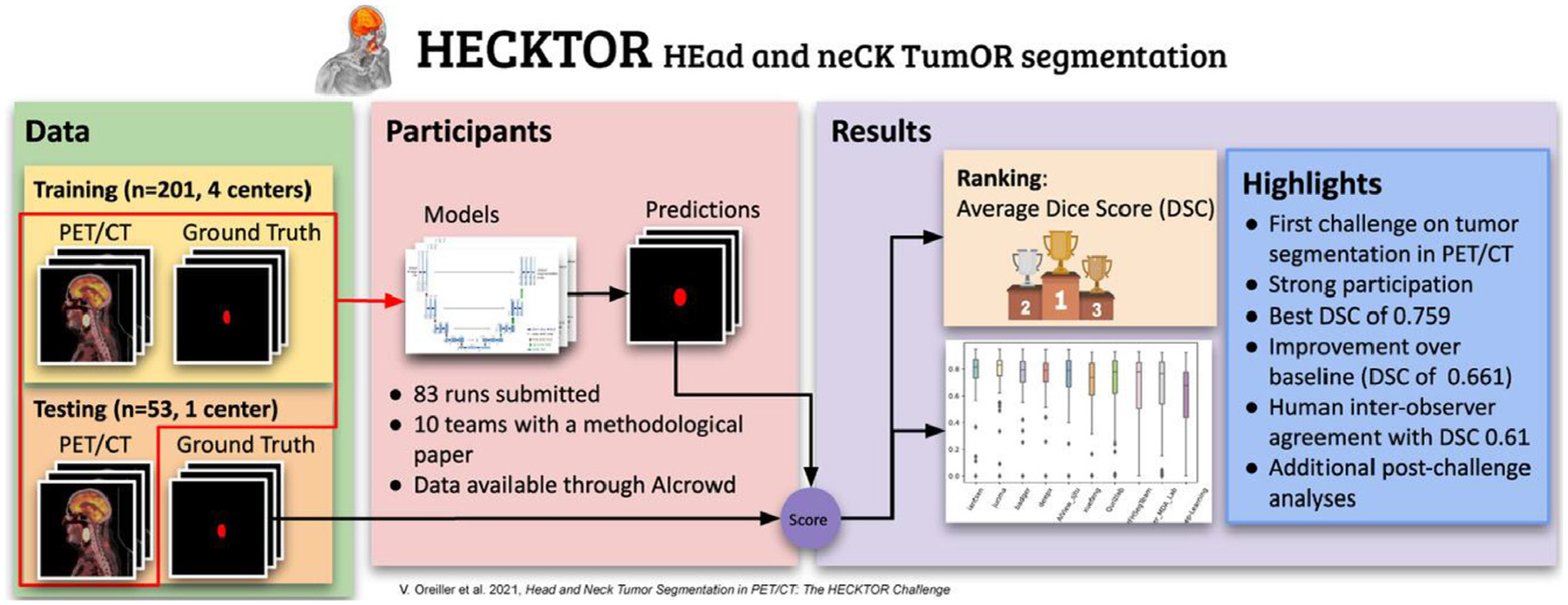
Overview of 2020 HEad and neCK TumOR (HECKTOR) challenge. Reprinted from Oreiller et al.^75^

More recently, the HEad and neCK TumOR (HECKTOR) challenge was established to benchmark the utility of computational methods using PET/CT imaging for head and neck cancer radiotherapy-related applications. Initiated in 2020 through the Medical Image Computing and Computer Assisted Intervention Society, the first edition of the challenge sought to develop automatic methods to segment primary gross tumor volumes in patients with oropharyngeal cancer (Fig. 8).^75^ The challenge utilized data from multiple Canadian and European medical institutions to provide participants with highly curated imaging and segmentation data. Participants were able to develop AI models, predominantly based on deep learning, to generate high-quality primary tumor segmentations on unseen test data. In the 2021 edition of the challenge, additional imaging data from a greater number of institutions were added to the training and testing datasets. Moreover, new tasks based on the prediction of progression-free survival were integrated into the challenge. While the official post-challenge analysis for the 2021 edition has not been made public yet, participants were shown to improve upon segmentation from the previous year’s challenge^76^ and demonstrate encouraging results to predict prognosis.^77,78^ A new edition of the challenge has been planned for 2022, which will include incorporating metastatic cervical lymph node segmentations and additional datasets from more institutions. The HECKTOR challenge is increasingly seen as a leader in current day radiation oncology related data challenges; we anticipate it will lead to important clinical innovations for translational AI approaches in coming years as its corresponding datasets continue to mature.

## Health Equity Considerations related to Public Datasets

While AI holds immense promise in improving the radiation oncology workflow through public medical imaging datasets, a thorough understanding of the current limitations of existing AI approaches is crucial before their widespread implementation. One such limitation is algorithmic bias/fairness, which if severe enough could further inequity and disparities in patient care. Algorithmic bias is not unique to advanced machine learning approaches. For example, a landmark paper by Obermeyer et al. demonstrated a widely used commercial algorithmic risk score based on simple demographic factors severely underestimated the health needs of the sickest marginalized groups (black patients) by focusing on financial costs.^79^ Subsequently, it is crucial to capture the biases in AI systems before they can be deployed in large-scale clinical settings. Unfortunately, most guiding principles for machine learning in healthcare applications do not directly address model fairness in detail.^80^ However, there is growing widespread interest in racial, gender, and socioeconomic disparities of AI-based healthcare algorithms.

Radiation oncology is not immune to biases in patient care, both at the level of the individuals,^81^ and systemically.^82^ It is well documented that marginalized racial groups often receive inferior care compared to wealthy or white patients,^83^ so the potential amplification of inequality caused by AI software in radiation oncology is a significant concern. While racial disparities have been modestly investigated for healthcare in AI generally, specific mechanisms for the existence of these disparities in imaging data/models remains relatively unexplored. However, a recent study by Banerjee et al.^84^ demonstrated that standard deep learning models could predict self-identified race from medical images with high performance. Importantly, they showed that this ability was not due to imaging-related surrogate covariates for race. Moreover, the performance of models persisted over a wide spectrum of clinical applications and image modalities, suggesting a significant and prevalent problem that warrants further investigation. These results are important since they suggest AI can trivially predict race where clinical experts cannot, thereby limiting human oversight and leading to potential downstream disparities, particularly for minorities.

**Figure 9.**
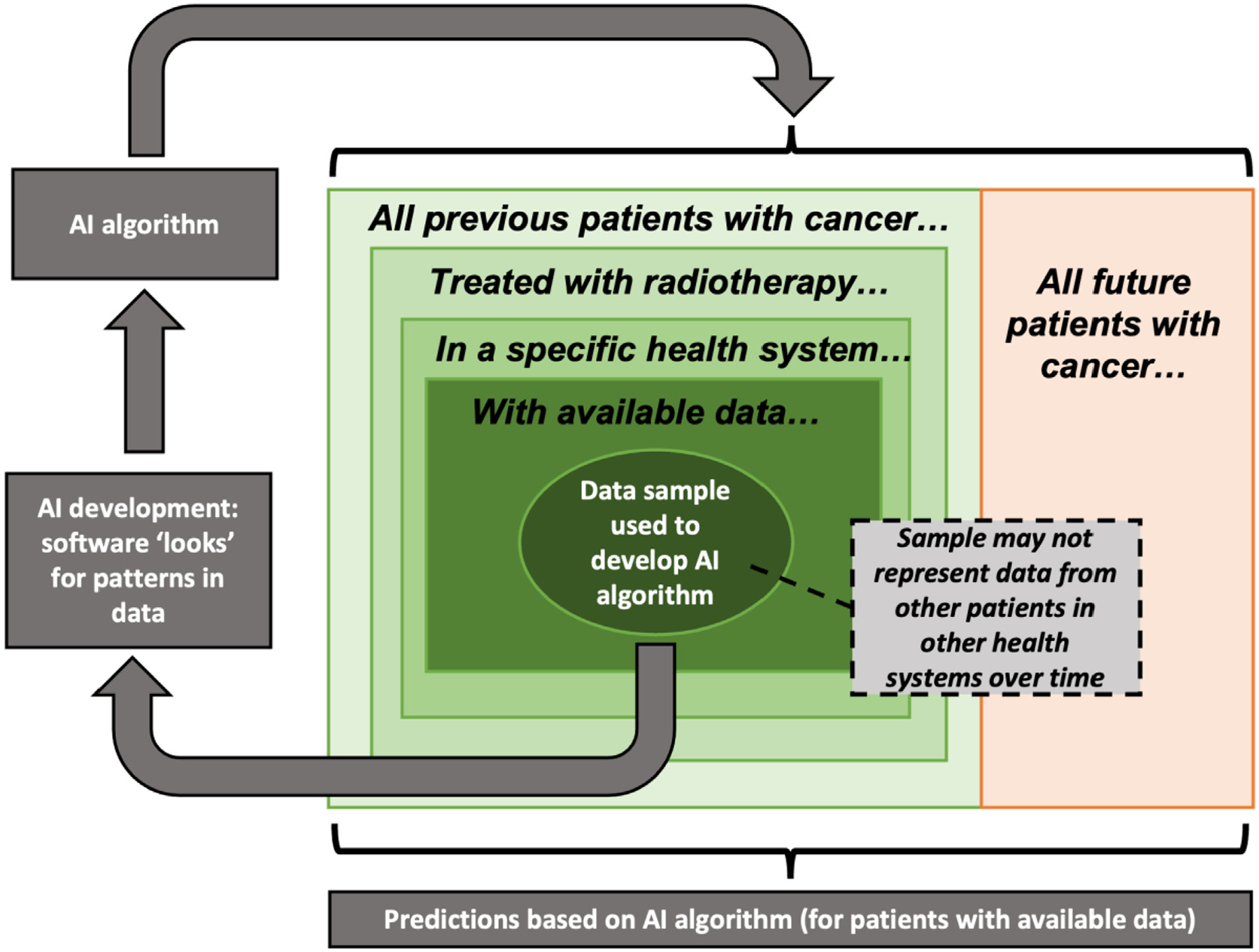
Statistical biases associated with AI predictions. Adapted from Chua et al.^85^

In an ideal setting, machine learning models should be trained and evaluated on data that accurately represent real-world data. In designing public data challenges, these concepts should be considered in the curation of training and test sets. Since data-driven methods inherently recognize patterns in training data, any bias already present in the data will be propagated to downstream models. Naturally, all datasets at some level will contain biases inherently tied to the sampling procedures. Importantly, biased sampling may lead to inaccurate predictions in unseen evaluation data, as illustrated in Figure 9. For example, data from public access clinical repositories are often disproportionately represented by Caucasian males. Ensuring representative sampling across time and data sources is an important method to reduce bias inherent to training data.^85^

Recommendations for operationalizing fairness for AI in medical data have been previously suggested,^80^ and these approaches should be subsequently implemented in imaging data for radiation oncology applications/data challenges. When curating datasets for data challenges or public dissemination, regardless of the target application, data on race, ethnicity, and socioeconomic status should also be collected and made available in order to assess their relationship to the underlying models where appropriate. Methods to circumvent algorithmic bias would include increases in model interpretability/explainability, either through inherently interpretable models or post-hoc techniques.^86^ Moreover, when developing and conceptualizing new AI models, it stands to reason that individuals with a vested interest in combating inequalities should be included in discussions to address potential sources and consequences of bias.^87^

## Conclusion

In summary, we describe how radiation oncology has benefited from FAIR scientific data distribution principles and will continue to benefit in the coming years. Given increasing attention by governing institutions and collaborative efforts, dissemination of radiation oncology data through structured repositories and public data challenges have led to algorithmic development and advancement, particularly with respect to AI-driven clinical decision support tools. A variety of concerns still plague the public dissemination of radiation oncology data, namely proper protection of patient PHI, ensuring standardized data objects and nomenclature, addressing health equity concerns, and consolidation of individual components of scientific dissemination. However, the future of public data distribution remains bright, and is certain to lead to continued innovation and clinical impact within the radiation oncology community.

## Figures and Tables

**Table 1:** Example of Variations in Standardized Nomenclatures Reported for Non-Target Structures by 16 Institutions

Structure	Number of Institutions	Examples
Left optic nerve	12	Lt Optic Nerve, OPTICN_L, OPTNRV_L, optic_nrv_l, L_optic_nerve, OPTIC_NRV_L, OpticNerve_L, LOPTIC, OpticNerve_L, Lef Optic Nerve, ON_L
Left lung	12	Lt Lung, Lung_L, LUNG_L, lung_l, L_lung, LLUNG, L Lung
Both lungs	12	Lungs, LUNGs, LUNG_TOTAL, lung_total, combined_lung, LUNG, LUNGS, Lung,BilatLung, Lung_Both
8th Cranial nerve	7	CN_VIII, cn_viii, CN8, CN_8
Right external iliac artery	2	A_ILIAC_E_R, a_iliac_e_r

Data adapted from American Association of Physicists in Medicine Task Group 263 Report.^33^

**Table 2:** HIPAA Data Elements That Encompass the “Safe Harbor” Method

Data Element	Description
Name	_-_
Address	All geographic subdivisions smaller than a state, including street address, city, county, precinct, ZIP code, and their equivalent geocodes, except for the initial 3 digits of the ZIP code if, according to the current publicly available data from the Bureau of the Census: The geographic unit formed by combining all ZIP codes with the same 3 initial digits contains more than 20,000 people; and The initial 3 digits of a ZIP code for all such geographic units containing 20,000 or fewer people is changed to 000.
Dates related to individual	birth date, admission date, discharge date, death date, and all ages over 89 and all elements of dates (including year) indicative of such age, except that such ages and elements may be aggregated into a single category of age 90 or older
Telephone numbers	-
Fax numbers	-
E-mail address	-
Social security number	-
Medical record number	-
Health plan beneficiary number	-
Account number	-
Certificate or license number	-
Vehicle/other device serial number	That is, license plate numbers
Device identifiers/serial numbers	-
Universal resource locators	That is, web URLs
Internet Protocol address	-
Biometric identifiers	Including finger- and voice- prints
Photographic image	Full-face photographs and any comparable images
Misc.	Any other characteristic that could uniquely identify the individual

**Table 3:** Current National Institute of Health (NIH)-Supported Domain-Specific Data Repositories Related to Radiation Oncology Research

Repository	Description
The cancer imaging archive (TCIA)	TCIA is a service which de-identifies and hosts a large archive of medical images of cancer accessible for public download. The data are organized as “Collections”, typically patients related by a common disease (e.g., lung cancer), image modality (MRI, CT, etc.) or research focus. DICOM is the primary file format used by TCIA for image storage. Supporting data related to the images such as patient outcomes, treatment details, genomics, pathology, and expert analyses are also provided when available.
Metabolomics work-bench (MetWB)	The Metabolomics Program’s Data Repository and Coordinating Center (DRCC), housed at the San Diego Supercomputer Center (SDSC), University of California, San Diego, has developed the Metabolomics Workbench. MetWB will serve as a national and international repository for metabolomics data and metadata and will provide analysis tools and access to metabolite standards, protocols, tutorials, training, and more.
Cancer nanotechnology laboratory (caNanoLab)	caNanoLab is a data sharing portal designed to facilitate information sharing in the biomedical nanotechnology research community to expedite and validate the use of nanotechnology in bio-medicine. caNanoLab provides support for the annotation of nanomaterials with characterizations resulting from physico-chemical, in vitro, and in vivo assays and the sharing of these characterizations and associated nanotechnology protocols in a secure fashion.
Genomic data commons (GDC)	The mission of the GDC is to provide the cancer research community with a unified data repository that enables data sharing across cancer genomic studies in support of precision medicine. The GDC contains clinical, biospecimen, and molecular data from several cancer research programs.
Proteomic data commons (PDC)	The Proteomic Data Commons hosts mass spectra and process data from cancer proteomic experiments. Many datasets have corresponding genomic and/or imaging data available in other nodes of the Cancer Research Data Commons.
The pediatric genomic data inventory (PGDI)	PGDI is an open-access resource for identifying and locating genomic datasets that can be used to further the understanding of childhood cancers and develop better treatment protocols for sick children. This resource lists ongoing and completed molecular characterization projects of pediatric cancer cohorts from the United States and other countries, along with some basic details and reference metadata. To contribute data to the PGDI, researchers need to create a secure submitter account through the PGDI Contributor Application at https://ocg.cancer.gov/programs/target/pgdi/contributor-application. The PGDI catalog will be continually updated as new information is deposited by the research community.
FaceBase	FaceBase is a NIDCR-funded data hub that hosts variety of data generated through dental, oral, and craniofacial research using model organisms and humans. The data offer spotlights high-throughput genetic, molecular, biological, imaging and computational techniques, as well as the database of 3D Facial Norms, developmental atlases and the Ontology of Craniofacial Development and Malformation (OCDM), Human Genome Analysis Interface (HGAI) and other resources.
ClinicalTrials.gov	ClinicalTrials.gov is a registry and results database of publicly and privately supported clinical studies of human participants conducted around the world.

Repositories and descriptions were derived from NIH Data Sharing Repositories online page (https://www.nlm.nih.gov/NIHbmic/domain_specific_repositories.html) on February 20, 2022.

**Table 4:** Currently Published Data Collections on The Cancer Imaging Archive (TCIA) Related to Radiation Oncology Applications

Collection	Location	Subjects	Image Types	Supporting Data
Vestibular-Schwannoma-SEG	Ear	242	MR, RTSTRUCT, RTPLAN, RTDOSE	Image Analyses
HNSCC	Head-Neck	627	CT, PT, MR, RTSTRUCT, RTPLAN, RTDOSE	Clinical, Image Analyses
NSCLC-Cetuximab (RTOG-0617)	Lung	490	CT, RTSTRUCT, RTDOSE, RTPLAN	Clinical
TCGA-HNSC	Head-Neck	227	CT, MR, PT, RTSTRUCT, RTPLAN, RTDOSE, Pathology	Clinical, Genomics
HNSCC-3DCT-RT	Head-Neck	31	CT, RTSTRUCT, RTDOSE	Image Analyses
Head-Neck-PET-CT	Head-Neck	298	PT, CT, RTSTRUCT, RTPLAN, RTDOSE	Clinical, Image Analyses, Software/Source Code
NRG-1308 (RTOG 1308)	Lung	12	CT, RTSTRUCT, RTPLAN, RTDOSE	Image Analyses
Head-Neck Cetuximab (RTOG 0522)	Head-Neck	111	CT, PT, RTSTRUCT, RTPLAN, RTDOSE	Image Analyses

Data were generated from TCIA collections page (https://www.cancerimagingarchive.net/collections/) using RTDOSE or RTPLAN as filtering criteria on February 18, 2022.
